# Body Fat Distribution and Systolic Blood Pressure in 10,000 Adults with Whole‐Body Imaging: UK Biobank and Oxford BioBank

**DOI:** 10.1002/oby.22509

**Published:** 2019-05-13

**Authors:** Deborah Malden, Ben Lacey, Jonathan Emberson, Fredrik Karpe, Naomi Allen, Derrick Bennett, Sarah Lewington

**Affiliations:** ^1^ Clinical Trial Service Unit and Epidemiological Studies Unit, Nuffield Department of Population Health University of Oxford Oxford UK; ^2^ Medical Research Council Population Health Research Unit Nuffield Department of Population Health, University of Oxford Oxford UK; ^3^ Oxford Centre for Diabetes, Endocrinology, and Metabolism University of Oxford Oxford UK; ^4^ Oxford Biomedical Research Centre, National Institute for Health Research Oxford University Hospitals Foundation Trust Oxford UK; ^5^ Cancer Epidemiology Unit University of Oxford Oxford UK

## Abstract

**Objective:**

This study aimed to quantify the associations of regional fat mass and fat‐free mass with systolic blood pressure.

**Methods:**

This analysis combined individual participant data from two large‐scale imaging studies: UK Biobank and Oxford BioBank. In both studies, participants were interviewed and measured, and they underwent dual‐energy x‐ray absorptiometry imaging. Linear regression was used to relate systolic blood pressure to anthropometric measures of adiposity (BMI, waist circumference, and waist to hip ratio) and dual‐energy x‐ray absorptiometry–derived measures of body composition (visceral android fat, subcutaneous android fat, subcutaneous gynoid fat, and fat‐free mass).

**Results:**

Among 10,260 participants (mean age 49; 96% white), systolic blood pressure was positively associated with visceral android fat (3.2 mmHg/SD in men; 2.8 mmHg/SD in women) and fat‐free mass (1.92 mmHg/SD in men; 1.64 mmHg/SD in women), but there was no evidence of an association with subcutaneous android or gynoid fat. Associations of systolic blood pressure with BMI were slightly steeper than those with waist circumference or waist to hip ratio; these associations remained unchanged following adjustment for fat‐free mass, but adjustment for visceral android fat eliminated associations with waist circumference and waist to hip ratio and more than halved associations with BMI.

**Conclusions:**

This analysis indicates that visceral fat is the primary etiological component of excess adiposity underlying the development of adiposity‐related hypertension.

## Introduction

Adiposity is an established risk factor for vascular disease, and this association is known to be mediated in part by raised blood pressure. Randomized controlled trials of weight loss interventions (and Mendelian randomization studies of blood pressure in relation to adiposity‐related genetic variants) support the causality of the relationship between adiposity and blood pressure 1, 2, 3, but the underlying mechanisms are not fully understood. In particular, the relevance of regional fat mass to blood pressure remains unclear.

Previous studies of adiposity and blood pressure have mostly used indirect measures of adiposity, such as BMI, waist circumference, or waist to hip ratio. The etiological relevance of general adiposity (as measured by BMI) and central adiposity (as measured by waist circumference or waist to hip ratio) has been inferred from these findings. Overall, BMI has been found to be more strongly related to blood pressure than measures of central adiposity 4, 5. In Western populations, BMI that is 1 kg/m^2^ higher is typically associated with approximately 1‐mmHg higher systolic blood pressure 6. However, these different measures of adiposity are highly correlated and are associated with differing levels of measurement error that affect the strength of the observed associations 7.

Measures of body composition that directly quantify regional fat distribution (such as dual‐energy x‐ray absorptiometry [DXA] imaging scans) can overcome some of these limitations. However, it has only recently become feasible to conduct large‐scale studies with whole‐body imaging scans. Evidence for the associations between regional body composition and blood pressure is, therefore, limited to a small number of studies, none of which was sufficiently large to reliably estimate the independent effect of different fat deposits on blood pressure levels 8, 9, 10, 11, 12.

This study examines the cross‐sectional associations of DXA‐derived measures of regional fat mass with systolic blood pressure, known to be more strongly predictive of vascular disease than diastolic blood pressure 13, using two large‐scale imaging studies (UK Biobank and Oxford BioBank) 14, 15. It aims to investigate the etiological relevance of regional fat mass to systolic blood pressure levels and to assess the extent to which the observed associations of systolic blood pressure with commonly used anthropometric measures of adiposity (BMI, waist circumference, and waist to hip ratio) are explained by their association with various regional fat depots.

## Methods

### UK Biobank imaging substudy

In 2006 through 2010, 503,000 adults (aged 40‐70 years) were recruited from the general population in the United Kingdom into a prospective cohort study 15. The UK Biobank aims to scan 100,000 participants by the end of 2023; the current analysis includes 5,170 of these participants who were included in the imaging pilot study (2014‐2015), which involved body composition assessments using whole‐body DXA imaging scans (Lunar iDXA densitometer; GE Healthcare, Chicago, Illinois). At the imaging assessment visit, information was collected on a range of demographic and lifestyle factors, including ethnicity, education, occupation, alcohol consumption, smoking status, and physical activity. Various measurements were also taken, including height, weight, waist and hip circumferences, and blood pressure. Height was measured with a nonstretchable tape, and weight was measured with digital scales (Tanita BC‐418MA body analyzer; Tanita Corporation of America, Inc., Arlington Heights, Illinois). Waist and hip circumferences were measured by using flexible plastic tape with the participant in the resting‐standing position. Systolic blood pressure and diastolic blood pressure were measured twice after the participant had been at rest for at least 5 minutes in the seated position by using a digital sphygmomanometer (Omron 705 IT; OMRON Healthcare Europe B.V., Hoofddorp, Netherlands) with a suitably sized cuff; the average of the two systolic blood pressure measures was used in all analyses.

### Oxford BioBank study

In 1999, the Oxford BioBank study recruited 5,425 adults (aged 29‐55 years) from the general population of Oxfordshire, UK 14. Participants were selected randomly from the National Health Service register and invited to undertake a whole‐body DXA imaging scan (Lunar iDXA). As in the UK Biobank, participants provided information on demographic factors, lifestyle, and medical history and had measurements taken, including height, weight, and waist and hip circumferences. Systolic blood pressure and diastolic blood pressure were measured four times by using a digital sphygmomanometer (Omron M3; OMRON Healthcare Europe B.V.) with the participants at rest in the seated position for at least 5 minutes. The mean of the final three systolic blood pressure measures was used in the analyses.

### DXA imaging

In both studies, DXA imaging scans were used to estimate fat mass in four mutually exclusive regions of the body: visceral android fat, subcutaneous android fat, subcutaneous gynoid fat, and other fat mass. Android, gynoid, and visceral fat were provided as automated output from the device, as estimated by using proprietary algorithms 16, 17, 18. The android region corresponds to roughly the abdomen and is defined by transverse planes at the top of the pelvis (iliac crest) and at 20% of the distance from the iliac crest to the top of the trunk. The gynoid region corresponds to the area around the hips and is defined by transverse planes at 1.5 and 3.5 times the height of the android region below the iliac crest. Subcutaneous android fat and all other fat were derived from these standard regions as additional mutually exclusive compartments of body fat.

### Statistical analysis

A meta‐analysis of the individual results from the UK Biobank and Oxford BioBank was performed. After excluding those with missing or extreme values of adiposity measures or systolic blood pressure (*n* = 335), 10,260 participants from both studies were available for analysis (Supporting Information Table S1). All analyses were performed separately for men and women 19 and were adjusted for age (5‐year groups) and socioeconomic factors (educational level [six groups] was used in the UK Biobank and occupation level [five groups] was used in the Oxford BioBank). The interrelationships between DXA‐derived measures of regional body fat and commonly used anthropometric measures of adiposity (BMI, waist circumference, and waist to hip ratio) were described by using the Pearson partial correlation coefficient (*r*). Linear regression was used to estimate the mean differences in each DXA‐derived measure of body composition (regional fat mass and total fat‐free mass) per 1‐SD difference in the levels of each anthropometric measure of adiposity.

After excluding participants on blood pressure–lowering therapy (*n* = 657), linear regression was also used to relate systolic blood pressure to each DXA‐derived measure of body composition (with mutual adjustment for the other DXA‐derived measures) and to each anthropometric measure of adiposity (with and without further adjustment for selected DXA‐derived measures of body composition); these analyses were further adjusted for height (sex‐specific fifths) and alcohol consumption (five groups) based on theoretical importance and significant contributions to the model.

Because of measurement error (or within‐person variability over time), regression analyses that use measurements of anthropometry on a single occasion may systematically underestimate the strength of the associations between anthropometric variables and other factors such as blood pressure (a phenomenon known as “regression dilution bias”) 20. As such, associations were corrected for regression dilution bias by dividing the beta coefficient of the association by the regression dilution ratio, as estimated by the correlation (*r*) between repeat measures at imaging and resurvey (2.1 years prior to imaging) in 2,063 UK Biobank participants (Supporting Information Table S2). This technique has been described in detail elsewhere 21. Associations corrected for regression dilution bias were described as association with “usual” (i.e., long‐term average) adiposity levels. Similarly, the usual SD of each anthropometric measure was obtained by multiplying the measured SD by √*r*.

Sensitivity analyses were conducted to assess the effect of blood pressure–lowering medication on the findings and to assess the effect of potential confounding by other lifestyle factors, such as smoking and physical activity. Analyses were also repeated by using log‐transformed, DXA‐derived variables. Ethics approval was obtained from the Oxford Ethics Committee and the Ethics and Governance Council for the Oxford BioBank study. The UK Biobank study received ethics approval from the National Information Governance Board for Health and Social Care and the National Health Service North West Multicentre Research Ethics Committee. All participants provided written informed consent. All analyses were conducted by using SAS version 9.3 (SAS Institute, Inc., Cary, North Carolina) and R version 3.3.1 (R Foundation for Statistical Computing, Vienna, Austria).

## Results

After exclusions, 10,260 participants contributed to the main analyses (Table 1). The mean age was 49 (SD 9.8) years, and 55% of participants were women. On average, participants in the UK Biobank imaging substudy were about 20 years older than those in the Oxford BioBank study (Supporting Information Table S3). Almost all (96%) participants were of white ethnicity, and more than half of participants had a professional occupation in both sexes. Among men, 8% were current smokers and 27% consumed alcohol daily, whereas among women, 6% were current smokers and 13% consumed alcohol daily.

**Table 1:** Baseline characteristics in Oxford BioBank and UK Biobank imaging studies combined, by sex

	Men (*n* = 4,619)	Women (*n* = 5,641)
**Demographics**		
**Age, mean (SD), y**	49.2 (10.1)	48.0 (9.5)
**White, *n* (%)**	4,448 (96)	5,419 (96)
**Professional occupation, *n* (%)**	2,966 (64)	3,170 (56)
**Current smoker, *n* (%)**	368 (8)	347 (6)
**Daily drinker, *n* (%)**	1,229 (27)	722 (13)
**Anthropometry, mean (SD)**		
**Height, cm**	177.5 (6.8)	164.1 (6.4)
**Weight, kg**	84.1 (13.2)	68.9 (12.7)
**BMI**	26.7 (3.8)	25.6 (4.6)
**Waist circumference, cm**	93.1 (10.2)	82.3 (11.6)
**Hip circumference, cm**	101.6 (6.9)	101.2 (9.4)
**Waist to hip ratio**	0.91 (0.06)	0.81 (0.07)
**DXA measures, mean (SD)**		
**Fat‐free mass, kg**	59.9 (7.2)	42.9 (5.2)
**Fat mass, kg**	23.8 (8.4)	25.6 (9.3)
**Visceral android fat**	1.4 (0.9)	0.6 (0.5)
**Subcutaneous android fat**	1.0 (0.5)	1.4 (0.7)
**Subcutaneous gynoid fat**	3.5 (1.2)	4.6 (1.5)
**Other fat**	17.8 (6.2)	19.0 (6.9)
**Blood pressure, mean (SD)**		
**Systolic blood pressure, mmHg**	134.0 (16.0)	124.5 (18.0)
**Diastolic blood pressure, mmHg**	79.8 (9.5)	75.3 (9.7)

Those with missing or out‐of‐range anthropometry, DXA, or blood pressure measures were excluded (Supporting Information Table S1).

DXA, dual‐energy x‐ray absorptiometry.

On average, men had higher BMI, waist circumference, and waist to hip ratio than women. The mean difference in weight between men and women (approximately 15 kg) was almost entirely due to differences in fat‐free mass. The distribution of body fat also differed between the sexes, with men having on average less subcutaneous fat in android and gynoid regions but more than twice the amount of visceral android fat (1.4 kg in men vs. 0.6 kg in women).

BMI was strongly correlated with waist circumference (*r* = 0.87 in men and 0.86 in women), and both measures were less strongly correlated with waist to hip ratio (*r* = 0.45‐0.80; Supporting Information Table S4). For DXA‐derived measures of body composition, subcutaneous android fat was strongly correlated with subcutaneous gynoid fat (*r* = 0.81 in men and 0.85 in women), and there were more modest correlations of these variables with visceral android fat (*r* = 0.46‐0.72). Fat‐free mass was only weakly correlated with DXA‐derived measures of regional body fat mass (*r* = 0.28‐0.53).

In both sexes, BMI, waist circumference, and waist to hip ratio were positively associated with DXA‐derived measures of regional body fat and fat‐free mass (Table 2; Supporting Information Table S5). The strengths of the associations were similar for each of the anthropometric measures with visceral android fat and subcutaneous android fat in men. The associations for other DXA‐derived measures of body composition were broadly similar for BMI and waist circumference but were somewhat shallower for waist to hip ratio.

**Table 2:** Mean difference in DXA‐derived body fat compartments per SD higher usual levels of BMI, waist circumference, and waist to hip ratio, by sex

Adiposity measure	Men (*n* = 4,619)			Women (*n* = 5,641)		
	SD	Mean difference, kg	95% CI	SD	Mean difference, kg	95% CI
**BMI**	3.7			4.5		
**Visceral android fat **	…	0.74	0.72‐0.76	…	0.44	0.43‐0.46
**Subcutaneous android fat **	…	0.33	0.32‐0.34	…	0.62	0.61‐0.70
**Subcutaneous gynoid fat **	…	1.00	0.98‐1.02	…	1.29	1.26‐1.31
**Other fat**	…	5.56	5.47‐5.64	…	6.53	6.46‐6.59
**Fat‐free mass**	…	4.11	3.95‐4.28	…	2.83	2.72‐2.94
**Waist circumference**	9.5			10.7		
**Visceral android fat **	…	0.73	0.72‐0.75	…	0.43	0.42‐0.44
**Subcutaneous android fat **	…	0.38	0.37‐0.39	…	0.64	0.63‐0.65
**Subcutaneous gynoid fat **	…	1.09	1.07‐1.11	…	1.19	1.16‐1.22
**Other fat**	…	6.10	6.02‐6.19	…	6.56	6.47‐6.65
**Fat‐free mass**	…	4.08	3.90‐4.27	…	2.97	2.85‐3.09
**Waist to hip ratio**	0.05			0.06		
**Visceral android fat **	…	0.72	0.70‐0.75	…	0.36	0.35‐3.38
**Subcutaneous android fat **	…	0.28	0.27‐0.30	…	0.39	0.37‐0.41
**Subcutaneous gynoid fat **	…	0.79	0.76‐0.83	…	0.43	0.38‐0.48
**Other fat**	…	5.17	4.99‐5.34	…	3.99	3.78‐4.19
**Fat‐free mass**	…	2.04	1.79‐2.29	…	1.47	1.31‐1.63

Exclusions are in Table 1.

DXA, dual‐energy x‐ray absorptiometry.

**Figure 1:**
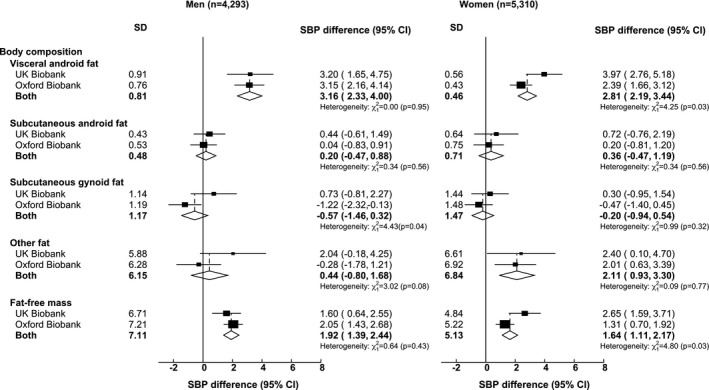
Associations of dual‐energy x‐ray absorptiometry (DXA)–derived measures of body composition with systolic blood pressure (SBP). Analyses are of the combined Oxford BioBank and UK Biobank imaging studies. Mean difference in SBP is given per SD higher DXA‐derived measure of body composition, with adjustment for age, height, education, occupation, alcohol consumption, and all other mutually exclusive DXA‐derived measures of fat and fat‐free mass. Exclusions are in Table 1. In addition, those on blood pressure–lowering medication were excluded.

Figure 1 shows the sex‐ and study‐specific associations of DXA‐derived measures of body composition with systolic blood pressure after adjustment for all other mutually exclusive DXA‐derived measures. Systolic blood pressure was positively associated with visceral android fat in both sexes (3.16 mmHg/SD in men and 2.81 mmHg/SD in women) and, to a lesser extent, with fat‐free mass (1.92 mmHg/SD in men and 1.64 mmHg/SD in women). In contrast, there was no evidence that either subcutaneous android fat or gynoid fat was associated with systolic blood pressure. Analyses of total leg fat instead of gynoid fat gave similar results (results not shown). “Other fat” was positively associated with systolic blood pressure in women only (2.11 mmHg/SD).

These associations were not materially changed by including participants who took blood pressure–lowering medication (Supporting Information Figure S1) or by further adjusting for smoking status and physical activity (Supporting Information Figure S2). With the exception of slightly shallower associations between visceral android fat and systolic blood pressure among women, log transformation of each body composition variable did not significantly impact associations (Supporting Information Figure S3). Overall, there was little evidence of significant heterogeneity in the findings between studies.

**Figure 2:**
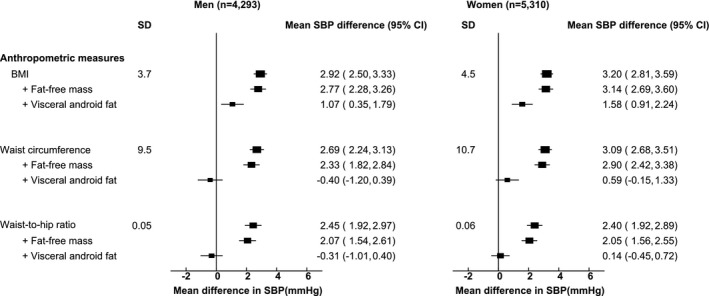
Associations of usual levels of BMI, waist circumference, and waist to hip ratio with systolic blood pressure (SBP), with and without adjustment for fat‐free mass and visceral android fat. Analyses are of the combined Oxford BioBank and UK Biobank imaging studies. The mean difference in SBP is given per SD higher usual level of each anthropometric variable, with adjustment for age, education, occupation, and alcohol consumption. Exclusions are in Table 1. In addition, those on blood pressure–lowering medication were excluded.

For both men and women, systolic blood pressure was positively related to each of the anthropometric measures of adiposity (Figure 2). These associations were largely unaltered after adjustment for fat‐free mass, but further adjustment for visceral android fat more than halved the associations with BMI and almost completely eliminated the associations with waist circumference and waist to hip ratio. Additional adjustment for other fat depots did not significantly alter these associations.

## Discussion

This report on two large‐scale imaging studies quantifies the associations of systolic blood pressure with regional fat mass and fat‐free mass. In both men and women, systolic blood pressure was positively associated with visceral fat and fat‐free mass, but there was no evidence of an association with subcutaneous android fat or gynoid fat. These results align with a growing body of genetic and observational evidence that has confirmed the adverse cardiometabolic effects of higher abdominal fat distribution 22, 23, 24, 25. Furthermore, the results indicate that much of the relation between systolic blood pressure and commonly used anthropometric measures of adiposity are explained by their correlation with visceral fat.

Several previous imaging studies (mostly using DXA or magnetic resonance imaging scans) have described cross‐sectional associations between blood pressure and regional fat mass or fat‐free mass 8, 9, 10, 11, 12. Consistent with the present report, these studies have described strong positive associations with visceral fat, but the findings for subcutaneous fat and lean body mass are more variable, with some describing positive associations 9, 10, and others describing negative 8, 11, 12. Most studies have made some adjustment for total fat mass but not for the range of mutually exclusive depots of fat and fat‐free mass, as in the present study. Furthermore, the size of these studies did not allow the strengths of these associations to be quantified reliably.

The relation between systolic blood pressure and the anthropometric measures of adiposity used in the present report have been well described in previous studies 5, 26, 27, 28, 29, 30. The strengths of the associations of systolic blood pressure with BMI and waist circumference in the present report are similar to those described in other populations of European origin; studies have indicated that these associations may be stronger in East Asian populations 5. In contrast, the associations with waist to hip ratio are somewhat stronger than those in most previous studies, perhaps reflecting the effect of correction for measurement error in this study, which was greater for waist to hip ratio than for the other measures and which has been rarely accounted for in other studies. Similarly, the few studies that have reported the relation between anthropometric measures of adiposity and regional fat mass, without correction for measurement error, have reported stronger associations of visceral fat mass with BMI and waist circumference than with waist to hip ratio. In the present study, however, the strengths of the associations with visceral fat were similar for each of the anthropometric measures of adiposity, but the associations with some of the other body components were notably weaker with waist to hip ratio (i.e., waist to hip ratio appears to be a more specific marker of visceral fat than these other adiposity measures).

The associations of systolic blood pressure with each of the anthropometric measures of adiposity were strongly attenuated when adjusting for visceral android fat, with complete attenuation of the associations with central adiposity (waist circumference and waist to hip ratio). This indicates that the relation between central adiposity and systolic blood pressure is mediated largely by visceral android fat (or some correlate of visceral fat) and suggests that there are additional mechanisms by which general adiposity (as measured by BMI) may influence blood pressure. Visceral fat is known to have distinct biological properties compared with body fat stored elsewhere, but the mechanisms by which it may influence systolic blood pressure are not clear; potential mechanisms involve abnormal renin‐angiotensin‐aldosterone regulation and sympathetic nervous system activation 31, 32, 33, 34. The hypothesized mechanisms by which general adiposity may influence blood pressure, in addition to its effects shared by central adiposity, include peripheral vascular resistance or renal sodium retention 5, 29, 35, 36. Some previous studies have suggested that menopause may increase the cardiometabolic consequences of obesity 20, and this may account for the slightly stronger association among women in the UK Biobank compared with the Oxford BioBank. The positive relation between fat‐free mass and systolic blood pressure was unexpected, but it has been reported in other populations 37.

The key strengths of the present study include its large sample size; the high‐quality measurements of body composition, blood pressure, and potential confounders; and the repeated surveys in a subset of UK Biobank participants to allow correction for measurement error of anthropometric variables. However, the cross‐sectional study design limits the ability to infer causality. There were rigorous quality‐control procedures for the DXA machines and digital sphygmomanometers in both studies. Importantly, however, regional fat was estimated using proprietary algorithms, and although externally validated, different DXA models used by the two studies may have produced estimates of varying accuracy. Furthermore, DXA imaging technology is unable to quantify ectopic fat within internal organs (such as the heart and liver), of which others have shown stronger correlations with metabolic disease, independently of total visceral fat 38, 39. Also, the body fat compartments used in the present study may contain a mixture of adipose tissues with differing biological characteristics (e.g., deep layers of subcutaneous fat are considered to have similar properties to visceral fat), but it was not possible to assess this in the present report 40. The UK Biobank study has the potential to address some of these issues (e.g., detailed characterization of adiposity by using magnetic resonance imaging scans) and investigate the relation of body composition to vascular disease and other risk factors, such as diabetes and lipid fractions.

Some of the observed variability of anthropometric measures may reflect real temporal variation in addition to measurement error. However, similar self‐correlations were observed between baseline and imaging visits (approximately 7 years apart; Supporting Information Table S2) compared with resurvey and imaging visits (approximately 2 years apart), indicating that real long‐term variability is unlikely to account for much of the observed variability between measures. Also, there were no repeat measurements available from the Oxford BioBank, and it was assumed that the within‐person variability in UK Biobank participants was comparable with that of Oxford Biobank, whereas, ideally, repeat measurements in both studies would have been used 7.

## Conclusion

Adiposity is an established risk factor for vascular disease, and this association is considered to be largely mediated by blood pressure, insulin resistance, and cholesterol 41. The findings of this report support the primary importance of visceral fat (or some correlates thereof) in the relation between adiposity and blood pressure in contrast to an apparent lack of association with subcutaneous android fat and gynoid fat. It also found a strong relation between fat‐free mass and systolic blood pressure, which requires further investigation. The associations with visceral fat were similar for each of the anthropometric measures of adiposity, and this correlation with visceral fat largely explained the relation between these measures and systolic blood pressure. Overall, these findings suggest that visceral fat is the primary etiological component of both central and general adiposity, underlying the development of adiposity‐related hypertension, and this has potential implications for enhanced risk stratification, prevention, and treatment of cardiovascular disease.

## References

[1] Bohula EA , Wiviott SD , McGuire DK , et al; CAMELLIA–TIMI 61 Steering Committee and Investigators . Cardiovascular safety of lorcaserin in overweight or obese patients. N Engl J Med 379:1107‐1117. 10.1056/NEJMoa1808721 30145941

[2] Fall T , Hagg S , Mägi R , et al; European Network for Genetic and Genomic Epidemiology (ENGAGE) consortium . The role of adiposity in cardiometabolic traits: a Mendelian randomization analysis. PLoS Med 2013;10:e1001474. doi:10.1371/journal.pmed.1001474 23824655 PMC3692470

[3] Holmes MV , Lange LA , Palmer T , et al. Causal effects of body mass index on cardiometabolic traits and events: a Mendelian randomization analysis. Am J Hum Genet 2014;94:198‐208. 24462370 10.1016/j.ajhg.2013.12.014 PMC3928659

[4] Gnatiuc L , Alegre‐Diaz J , Halsey J , et al. Adiposity and blood pressure in 110 000 Mexican adults. Hypertension 2017;69:608‐614. 28223471 10.1161/HYPERTENSIONAHA.116.08791 PMC5344187

[5] Chen Z , Smith M , Du H , et al; China Kadoorie Biobank Collaborative Group . Blood pressure in relation to general and central adiposity among 500 000 adult Chinese men and women. Int J Epidemiol 2015;44:1305‐1319. 25747585 10.1093/ije/dyv012 PMC4588860

[6] Prospective Studies Collaboration ; Whitlock G , Lewington S , Sherliker P , et al. Body‐mass index and cause‐specific mortality in 900 000 adults: collaborative analyses of 57 prospective studies. Lancet 2009;373:1083‐1096. 19299006 10.1016/S0140-6736(09)60318-4 PMC2662372

[7] Emerging Risk Factors Collaboration ; Wormser D , Kaptoge S , Di Angelantonio E , et al. Separate and combined associations of body‐mass index and abdominal adiposity with cardiovascular disease: collaborative analysis of 58 prospective studies. Lancet 2011;377:1085‐1095. 21397319 10.1016/S0140-6736(11)60105-0 PMC3145074

[8] Chandra A , Neeland IJ , Berry JD , et al. The relationship of body mass and fat distribution with incident hypertension: observations from the Dallas Heart Study. J Am Coll Cardiol 2014;64:997‐1002. 25190234 10.1016/j.jacc.2014.05.057

[9] Ding J , Visser M , Kritchevsky SB , et al. The association of regional fat depots with hypertension in older persons of white and African American ethnicity. Am J Hypertens 2004;17:971‐976. 15485762 10.1016/j.amjhyper.2004.05.001

[10] Foy CG , Hsu FC , Haffner SM , et al. Visceral fat and prevalence of hypertension among African Americans and Hispanic Americans: findings from the IRAS family study. Am J Hypertens 2008;21:910‐916. 18566594 10.1038/ajh.2008.213 PMC2551313

[11] Vasan SK , Osmond C , Canoy D , et al. Comparison of regional fat measurements by dual‐energy X‐ray absorptiometry and conventional anthropometry and their association with markers of diabetes and cardiovascular disease risk. Int J Obes (Lond) 2018;42:850‐857. 29151596 10.1038/ijo.2017.289 PMC5965665

[12] Yano Y , Vongpatanasin W , Ayers C , et al. Regional fat distribution and blood pressure level and variability: the Dallas Heart Study. Hypertension 2016;68:576‐583. 27432862 10.1161/HYPERTENSIONAHA.116.07876 PMC4982814

[13] Wang JG , Staessen JA , Franklin SS , Fagard R , Gueyffier F . Systolic and diastolic blood pressure lowering as determinants of cardiovascular outcome. Hypertension 2005;45:907‐913. 15837826 10.1161/01.HYP.0000165020.14745.79

[14] Karpe F , Vasan SK , Humphreys SM , et al. Cohort profile: the Oxford Biobank. Int J Epidemiol 2018;47:21‐21g. doi:10.1093/ije/dyx132 29040543 PMC5837504

[15] Sudlow C , Gallacher J , Allen N , et al. UK Biobank: an open access resource for identifying the causes of a wide range of complex diseases of middle and old age. PLoS Med 2015;12:e1001779. doi:10.1371/journal.pmed.1001779 25826379 PMC4380465

[16] Ergun DL , Rothney MP , Oates MK , Xia Y , Wacker WK , Binkley NC . Visceral adipose tissue quantification using Lunar Prodigy. J Clin Densitom 2013;16:75‐78. 23148876 10.1016/j.jocd.2012.09.002

[17] Kaul S , Rothney MP , Peters DM , et al. Dual‐energy X‐ray absorptiometry for quantification of visceral fat. Obesity (Silver Spring) 2012;20:1544. 10.1038/oby.2011.393 PMC3361068 22282048

[18] Lee JJ , Freeland‐Graves JH , Pepper MR , Stanforth PR , Xu B . Prediction of android and gynoid body adiposity via a three‐dimensional stereovision body imaging system and dual‐energy X‐ray absorptiometry. J Am Coll Nutr 2015;34:367‐377. 25915106 10.1080/07315724.2014.966396 PMC5690984

[19] Faulkner JL , Belin de Chantemèle EJ . Sex differences in mechanisms of hypertension associated with obesity. Hypertension 2018;71:15‐21. 29133358 10.1161/HYPERTENSIONAHA.117.09980 PMC5730468

[20] Clarke R , Shipley M , Lewington S , et al. Underestimation of risk associations due to regression dilution in long‐term follow‐up of prospective studies. Am J Epidemiol 1999;150:341‐353. 10453810 10.1093/oxfordjournals.aje.a010013

[21] Clarke R , Emberson JR , Breeze E , et al. Biomarkers of inflammation predict both vascular and non‐vascular mortality in older men. Eur Heart J 2008;29:800‐809. 18303034 10.1093/eurheartj/ehn049

[22] Dahlman I , Rydén M , Brodin D , Grallert H , Strawbridge RJ , Arner P . Numerous genes in loci associated with body fat distribution are linked to adipose function. Diabetes 2016;65:433‐437. 26798124 10.2337/db15-0828

[23] Karpe F , Pinnick KE . Biology of upper‐body and lower‐body adipose tissue–link to whole‐body phenotypes. Nat Rev Endocrinol 2015;11:90‐100. 25365922 10.1038/nrendo.2014.185

[24] Lotta LA , Wittemans LBL , Zuber V , et al. Association of genetic variants related to gluteofemoral vs abdominal fat distribution with type 2 diabetes, coronary disease, and cardiovascular risk factors. JAMA 2018;320:2553‐2563. 30575882 10.1001/jama.2018.19329 PMC6583513

[25] Rydén M , Andersson DP , Bergström IB , Arner P . Adipose tissue and metabolic alterations: regional differences in fat cell size and number matter, but differently: a cross‐sectional study. J Clin Endocrinol Metab 2014;99:E1870‐E1876. 24937536 10.1210/jc.2014-1526

[26] Dorresteijn JAN , Visseren FLJ , Spiering W . Mechanisms linking obesity to hypertension. Obes Rev 2012;13:17‐26. 21831233 10.1111/j.1467-789X.2011.00914.x

[27] Gnatiuc L , Alegre‐Díaz J , Halsey J , et al. Adiposity and blood pressure in 110 000 Mexican adults. Hypertension 2017;69:608‐614. 28223471 10.1161/HYPERTENSIONAHA.116.08791 PMC5344187

[28] Huxley R , James WP , Barzi F , et al; Obesity in Asia Collaboration . Ethnic comparisons of the cross‐sectional relationships between measures of body size with diabetes and hypertension. Obes Rev 2008;9(suppl 1):53‐61. 18307700 10.1111/j.1467-789X.2007.00439.x

[29] Kotsis V , Stabouli S , Papakatsika S , Rizos Z , Parati G . Mechanisms of obesity‐induced hypertension. Hypertens Res 2010;33:386‐393. 20442753 10.1038/hr.2010.9

[30] Lee CM , Huxley RR , Wildman RP , Woodward M . Indices of abdominal obesity are better discriminators of cardiovascular risk factors than BMI: a meta‐analysis. J Clin Epidemiol 2008;61:646‐653. 18359190 10.1016/j.jclinepi.2007.08.012

[31] Grassi G , Dell'Oro R , Facchini A , Quarti Trevano F , Bolla GB , Mancia G . Effect of central and peripheral body fat distribution on sympathetic and baroreflex function in obese normotensives. J Hypertens 2004;22:2363‐2369. 15614031 10.1097/00004872-200412000-00019

[32] Landsberg L . Insulin‐mediated sympathetic stimulation: role in the pathogenesis of obesity‐related hypertension (or, how insulin affects blood pressure, and why). J Hypertens 2001;19:523‐528. 11327624 10.1097/00004872-200103001-00001

[33] Straznicky NE , Eikelis N , Lambert EA , Esler MD . Mediators of sympathetic activation in metabolic syndrome obesity. Curr Hypertens Rep 2008;10:440‐447. 18959829 10.1007/s11906-008-0083-1

[34] Moan A , Nordby G , Rostrup M , Eide I , Kjeldsen SE . Insulin sensitivity, sympathetic activity, and cardiovascular reactivity in young men. Am J Hypertens 1995;8:268‐275. 7794576 10.1016/0895-7061(94)00206-Q

[35] Kalil GZ , Haynes WG . Sympathetic nervous system in obesity‐related hypertension: mechanisms and clinical implications. Hypertens Res 2012;35:4‐16. 22048570 10.1038/hr.2011.173 PMC3902842

[36] Poirier P , Giles TD , Bray GA , et al; American Heart Association; Obesity Committee of the Council on Nutrition, Physical Activity, and Metabolism . Obesity and cardiovascular disease: pathophysiology, evaluation, and effect of weight loss: an update of the 1997 American Heart Association scientific statement on obesity and heart disease from the Obesity Committee of the Council on Nutrition, Physical Activity, and Metabolism. Circulation 2006;113:898‐918. 16380542 10.1161/CIRCULATIONAHA.106.171016

[37] Bastawrous MC , Piernas C , Bastawrous A , et al. Reference values for body composition and associations with blood pressure in Kenyan adults aged ≥50 years old. Eur J Clin Nutr 2019;73:558‐565. 29769749 10.1038/s41430-018-0177-z PMC6124645

[38] Fabbrini E , Magkos F , Mohammed BS , et al. Intrahepatic fat, not visceral fat, is linked with metabolic complications of obesity. Proc Natl Acad Sci U S A 2009;106:15430‐15435. 19706383 10.1073/pnas.0904944106 PMC2741268

[39] Speliotes EK , Massaro JM , Hoffmann U , et al. Fatty liver is associated with dyslipidemia and dysglycemia independent of visceral fat: the Framingham Heart Study. Hepatology 2010;51:1979‐1987. 20336705 10.1002/hep.23593 PMC3023160

[40] Marinou K , Hodson L , Vasan SK , et al. Structural and functional properties of deep abdominal subcutaneous adipose tissue explain its association with insulin resistance and cardiovascular risk in men. Diabetes Care 2014;37:821‐829. 24186879 10.2337/dc13-1353

[41] Danaei G , Finucane MM , Lin JK , et al; Global Burden of Metabolic Risk Factors of Chronic Diseases Collaborating Group (Blood Pressure) . National, regional, and global trends in systolic blood pressure since 1980: systematic analysis of health examination surveys and epidemiological studies with 786 country‐years and 5.4 million participants. Lancet 2011;377:568‐577. 21295844 10.1016/S0140-6736(10)62036-3

