# Supplementary material for: Body Fat Distribution and Systolic Blood Pressure in 10,000 Adults with Whole‐Body Imaging: UK Biobank and Oxford BioBank
Source: Obesity (Silver Spring). 2019 May 13;27(7):1200–6. doi: 10.1002/oby.22509 (PMC6618903; doi:10.1002/oby.22509)
Supplement: Supplementary file 1 [file OBY-27-1200-s001.pdf]

Body fat distribution and systolic blood pressure in 10,000 adults with whole-body imaging: UK Biobank and Oxford Biobank

Debbie Malden[1], Ben Lacey[1], Jonathan Emberson[1,2], Fredrik Karpe[3,4], Naomi Allen[1,5], Derrick Bennett[2], Sarah Lewington[1,2,4]

[1]Clinical Trial Service Unit & Epidemiological Studies Unit (CTSU), Nuffield Department of Population Health (NDPH), University of Oxford, UK; [2]MRC Population Health Research Unit, CTSU, NDPH, University of Oxford, UK; [3]The Oxford Centre for Diabetes, Endocrinology and Metabolism (OCDEM), University of Oxford, UK; [4] National Institute for Health Research (NIHR) Oxford Biomedical Research Centre (BRC), Oxford University Hospitals Foundation Trust, Oxford, UK; [5]Cancer Epidemiology Unit (CEU), University of Oxford, Oxford, UK

Supplementary file

Table/figure	Description
**Table S1**	Exclusion criteria for participants included in analyses, by study
**Table S2**	Correlation coefficients between anthropometric measurements at imaging visit and resurvey assessments approximately 2.1 and 7.2 years prior
**Table S3**	Baseline characteristics by study
**Table S4**	Adjusted correlation coefficients between selected body size and composition variables
**Table S5**	BMI, waist circumference and waist-to-hip ratio versus body composition, by sex
**Figure S1**	Mean change in systolic blood pressure (SBP) per 1 standard deviation (SD) higher DXA-derived body composition variable in 10,260 participants, by study and sex
**Figure S2**	Mean change in systolic blood pressure (SBP) per 1 standard deviation (SD) higher regional body composition after further adjustment for smoking and physical activity, by sex
**Figure S3**	Mean change in systolic blood pressure (SBP) per 1 standard deviation (SD) higher log-transformed body composition, by study and sex

Table S1. Exclusion criteria for participants of the Oxford Biobank (OBB) and UK Biobank (UKB) imaging studies

	Men (n= 4 708)						Women (n=5 887)						Total	
	Missing		Lower limit	Upper limit	Outliers		Missing		Lower limit	Upper limit	Outliers		OBB	UKB
	OBB	UKB			OBB	UKB	OBB	UKB			OBB	UKB		
**Directly measured**														
Body weight (kg)	0	0	<40kg	≥170kg	0	0	0	0	<40kg	≥150kg	2	3	2	3
Height (cm)	0	10	<140cm	≥200cm	6	0	4	11	<140cm	≥185cm	7	2	17	23
Waist circumference(cm)	2	10	<65cm	≥150cm	2	0	3	10	<55cm	≥140cm	5	0	12	20
Hip circumference (cm)	2	10	<80cm	≥145cm	1	1	4	10	<70cm	≥150cm	7	0	14	21
Body-mass index (kg/m^2)	0	10	<15	≥50	0	0	4	11	<15	≥60	5	2	9	23
Waist-to-hip ratio	3	10	<0.7	≥1.2	4	0	4	10	<0.60	≥1.2	6	0	17	20
**DXA measurements, kg**														
Body fat	0	0	<3kg	≥60kg	8	10	0	0	<3kg	≥60kg	26	8	34	18
Subcutaneous android fat	7	0	<0.01kg	≥5kg	7	1	11	0	<0.01kg	≥5kg	17	0	43	1
Visceral android fat	7	26	<0.01kg	≥5kg	15	11	11	35	<0.01kg	≥5kg	141	4	174	76
Subcutaneous gynoid fat	0	0	<0.1kg	≥15kg	0	0	0	0	<0.1kg	≥15kg	1	0	1	0
Other fat	7	0	<2kg	≥50kg	2	0	0	0	<2kg	≥50	10	0	19	0
**Systolic blood pressure**	2	11	<80mmHg	≥240mmHg	1	0	2	8	<80mm Hg	≥240mmHg	3	0	8	19
**Total excluded**													198	137
**Total included**													5227	5033
**Total combined**														10, 260

DXA =Dual energy X-ray Absorptiometry

Table S2. Correlation coefficients between anthropometry measures at imaging visit and resurvey assessments in the UK Biobank approximately 2.1 and 7.2 years prior

	Self-correlation between 1st resurvey and imaging visit (~2.1 years after)			Self-correlation between baseline survey and imaging visit (~7.2 years after)		
	Men (n=1003)	Women (n=1060)	All (n=2063)*	Men (n=2390)	Women (n=2643)	All (n=5033)*
**Height**	0.99	0.99	0.99	0.98	0.98	0.98
**Weight**	0.96	0.95	0.96	0.93	0.91	0.92
**Body mass index**	0.95	0.95	0.95	0.91	0.91	0.91
**Waist Circumference**	0.83	0.84	0.84	0.79	0.81	0.80
**Hip Circumference**	0.80	0.86	0.84	0.78	0.82	0.80
**Waist-to-hip ratio**	0.63	0.65	0.64	0.62	0.65	0.64

*Adjusted for sex. Those with missing or outlying anthropometry, DXA and blood pressure variables were excluded (see Supplementary Table 1).

Table S3. Baseline characteristics by study

	Oxford Biobank (N=5 227)		UK Biobank (n=5 033)	
	Men (n=2 229)	Women (n=2 998)	Men (n=2 390)	Women (n=2 643)
**Demographic characteristics**				
Mean age, years (SD)	41.5 (5.9)	41.8 (6.1)	63.2 (7.5)	61.8 (7.5)
White, n (%)	2 162 (97%)	2 878 (96%)	2 286 (96%)	2 541 (96%)
Professional occupation, n (%)	1 515 (68%)	1 808 (60%)	1 451 (61%)	1 371 (52%)
Current smoker, n (%)	240 (11%)	249 (8%)	128 (5%)	98 (4%)
Daily drinker, n (%)	670 (30%)	343 (11%)	559 (23%)	379 (14%)
**Anthropometry, mean (SD)**				
Height, cm	179.2 (6.6)	165.5 (6.2)	176 (6.6)	162 (6.3)
Weight, kg	85.3 (13.6)	69.3 (13.0)	83.1 (12.7)	68.5 (12.3)
BMI, kg/m^2	26.2 (3.9)	25.0 (4.6)	26.8 (3.6)	26.0 (4.5)
Waist circumference, cm	92.8 (10.9)	82.5 (11.9)	93.4 (9.4)	82.1 (11.2)
Hip circumference, cm	101.7 (7.1)	101.3 (9.3)	101.6 (6.8)	101.1 (9.4)
Waist-to-hip ratio	0.91 (0.07)	0.81 (0.07)	0.92 (0.05)	0.81 (0.06)
**DXA measures, mean (SD)**				
Fat-free mass, kg	61.4 (7.3)	43.9 (5.3)	58.5 (6.8)	41.8 (4.9)
Percentage body fat, %				
Fat mass, kg	22.9 (8.6)	24.7 (9.4)	24.6 (8.1)	26.7 (9.1)
Visceral fat	1.08 (0.76)	0.43 (0.43)	1.67 (0.91)	0.78 (0.57)
Subcutaneous android fat	1.10 (0.53)	1.43 (0.75)	0.98 (0.44)	1.42 (0.64)
Subcutaneous gynoid fat	3.40 (1.19)	4.52 (1.48)	3.54 (1.16)	4.65 (1.45)
Other fat	17.09 (6.27)	18.20 (6.92)	18.40 (5.96)	19.80 (6.67)
**Blood pressure, mean (SD)**				
Systolic, mmHg	127.2 (12.3)	116.6 (12.9)	140.3 (16.6)	133.4 (18.7)
Diastolic, mmHg	79.0 (9.2)	73.5 (9.2)	80.6 (9.8)	77.4 (9.9)

DXA = Dual energy X-ray Absorptiometry; Those with missing or outlying anthropometry, DXA and blood pressure variables were excluded (see Supplementary Table 1)

Table S4. Adjusted correlation coefficients between selected body size and composition variables

		Anthropometry variables		DXA variables				
		BMI	Waist-to-hip ratio	Fat-free mass	Visceral android fat	Subcutaneous android fat	Subcutaneous gynoid fat	Other fat
**Waist circumference**	Male:	0.87	0.80	0.54	0.80	0.71	0.83	0.90
	Female:	0.86	0.73	0.56	0.83	0.82	0.73	0.88
**BMI**	Male:		0.62	0.58	0.79	0.67	0.81	0.88
	Female:		0.45	0.56	0.77	0.85	0.85	0.93
**Waist-to-hip ratio**	Male:			0.24	0.67	0.47	0.52	0.66
	Female:			0.23	0.62	0.40	0.20	0.45
**Fat-free mass**	Male:				0.37	0.28	0.41	0.43
	Female:				0.39	0.49	0.52	0.53
**Visceral android fat**	Male:					0.46	0.72	0.87
	Female:					0.67	0.62	0.82
**Subcutaneous android fat**	Male:						0.81	0.79
	Female:						0.85	0.91
**Subcutaneous gynoid fat**	Male:							0.93
	Female:							0.91

DXA=Dual energy X-ray Absorptiometry; Analyses in 10,260 participants (exclusions as in Table 1); Correlation coefficients were adjusted for age (5-year groups).

Table S5. BMI, waist circumference and waist-to-hip ratio versus body composition, by sex

			Age- and education-adjusted means (95% CI)				
**Men (n=4,619)**	**Level**	**N**	**Visceral fat**	**Subcutaneous android fat**	**Subcutaneous gynoid fat**	**Other fat**	**Fat-free mass**
**BMI, kg/m^2**	22.01	923	0.58 (0.55- 0.60)	0.65 (0.63- 0.68)	2.33 (2.29- 2.37)	11.19 (10.99- 11.38)	54.68 (54.34- 55.02)
	24.51	925	0.97 (0.94- 1.00)	0.88 (0.86- 0.90)	2.93 (2.89- 2.96)	14.69 (14.51 -14.88)	57.50 (57.14- 57.87)
	26.29	923	1.29 (1.25- 1.32)	1.01 (0.99- 1.03)	3.32 (3.28- 3.37)	17.10 (16.90 - 17.29)	59.69 (59.31- 60.07)
	28.26	924	1.69 (1.66- 1.73)	1.17 (1.15- 1.19)	3.89 (3.84- 3.94)	20.20 (19.99- 20.41)	61.76 (61.38- 62.15)
	32.37	924	2.45 (2.40- 2.50)	1.53 (1.50- 1.57)	4.97 (4.89- 5.04)	26.06 (25.72- 26.41)	65.79 (65.33- 66.25)
**Waist circumference, cm**	79.75	902	0.54 (0.52- 0.57)	0.62 (0.60- 0.64)	2.28 (2.24- 2.32)	10.92 (10.73- 11.11)	55.21 (54.82- 55.61)
	87.20	843	0.95 (0.92- 0.97)	0.87 (0.83- 0.87)	2.88 (2.84- 3.92)	14.46 (14.27- 14.65)	57.78 (57.37- 58.20)
	91.92	996	1.27 (1.24- 1.30)	1.01 (0.99- 1.03)	3.33 (3.29- 3.36)	17.08 (16.91- 17.24)	59.33 (58.98 - 59.68)
	97.73	1005	1.70 (1.67- 1.73)	1.17 (1.15- 1.19)	3.89 (3.85- 3.94)	20.20 (20.01- 20.38)	61.47 (61.10- 61.84)
	108.58	873	2.49 (2.44- 2.54)	1.59 (1.56- 1.63)	5.10 (5.02- 5.17)	26.66 (26.32- 26.99)	65.68 (65.21 - 66.16)
**Waist-to-hip ratio**	0.83	924	0.65 (0.62- 0.68)	0.73 (0.70- 0.76)	2.65 (2.58- 2.72)	12.43 (12.13- 12.73)	57.93 (57.48- 58.39)
	0.88	930	1.01 (0.97 - 1.05)	0.95 (0.93- 0.98)	3.13 (3.07- 3.19)	15.54 (15.24 - 15.83)	58.76 (58.32- 59.19)
	0.91	923	1.32 (1.28- 1.36)	1.06 (1.04- 1.09)	3.47(3.41- 3.54)	17.56 (17.26- 17.85)	59.77 (59.34- 60.20)
	0.95	913	1.68 (1.64- 1.73)	1.15 (1.12- 1.18)	3.80 (3.73- 3.87)	19.89 (19.57- 20.21)	60.23 (59.78- 60.67)
	1.00	929	2.27 (2.22- 2.33)	1.37 (1.33- 1.41)	4.40 (4.32- 4.48)	23.89 (23.47- 24.30)	62.61 (62.10- 63.12)
**Women (n=5,641)**							
**BMI, kg/m^2**	20.339	1128	0.19 (0.19- 0.20)	0.71 (0.70- 0.73)	3.11 (3.07- 3.15)	11.64 (11.49- 11.79)	39.74 (39.50- 39.97)
	22.757	1128	0.31 (0.30- 0.32)	1.05 (1.03- 1.06)	3.78 (3.74- 3.82)	14.81 (14.67- 14.95)	41.49 (41.25- 41.74)
	24.739	1128	0.48 (0.47- 0.50)	1.35 (1.33- 1.37)	4.41 (4.37- 4.46)	17.87 (17.72- 18.02)	42.39 (42.14- 42.63)
	27.342	1129	0.72 (0.70- 0.74)	1.69 (1.66- 1.71)	5.11 (5.06- 5.17)	21.51 (21.34- 21.68)	43.75 (43.48- 44.01)
	32.876	1128	1.26 (1.23 - 1.29)	2.39 (2.36- 2.43)	6.53 (6.45- 6.61)	29.15 (28.84- 29.45)	47.27 (46.97- 47.56)
**Waist circumference, cm**	68.558	1171	0.19 (0.18- 0.20)	0.73 (0.71 - 0.75)	3.29 (3.25- 3.34)	12.10 (11.94- 12.26)	39.82 (39.58- 40.06)
	75.085	1043	0.31 (0.30- 0.32)	1.07 (1.05- 1.09)	3.89 (3.84- 3.94)	15.10 (14.93- 15.28)	41.57 (41.31- 41.83)
	80.441	1179	0.48 (0.47- 0.50)	1.34 (1.32- 1.36)	4.43 (4.37- 4.48)	17.87 (17.68- 18.06)	42.34 (42.10 - 42.57)
	87.143	1093	0.71 (0.69- 0.73)	1.68 (1.65- 1.70)	5.05 (4.98 - 5.11)	21.38 (21.15- 21.60)	43.58 (43.32- 43.83)
	100.102	1155	1.27 (1.24- 1.30)	2.36 (2.32- 2.40)	6.29 (6.20- 6.38)	28.49 (28.15- 28.83)	47.35 (47.06- 47.64)
**Waist-to-hip ratio**	0.723	1133	0.27 (0.26- 0.29)	1.08 (1.04- 1.11)	4.26 (4.18- 4.35)	15.56 (15.24- 15.87)	41.80 (41.51 - 42.08)
	0.77	1119	0.38 (0.36- 0.40)	1.18 (1.15- 1.22)	4.22 (4.14 - 4.30)	16.34 (16.01- 16.68)	42.19 (41.92 - 42.46)
	0.805	1131	0.51 (0.49- 0.53)	1.39 (1.35 - 1.42)	4.50 (4.42- 4.59)	18.33 (17.98- 18.68)	42.32 (42.05- 42.60)
	0.846	1130	0.72 (0.69- 0.74)	1.59 (1.55- 1.63)	4.79 (4.70- 4.88)	20.56 (20.17- 20.94)	43.34 (43.03- 43.64)
	0.914	1128	1.09 (1.05- 1.13)	1.93 (1.89- 1.98)	5.16 (5.06- 5.25)	24.08 (23.66 - 24.51)	45.06 (44.74- 45.38)

Means adjusted for age (5-year groups) and qualification attainment (5 groups). Analyses in 10, 260 imaging participants; exclusions as Supplementary Table 1.

Figure S1. Mean change in systolic blood pressure (SBP) per 1 standard deviation (SD) higher DXA-derived body composition variable in 10,260 participants including those on blood pressure lowering medication, by study and sex

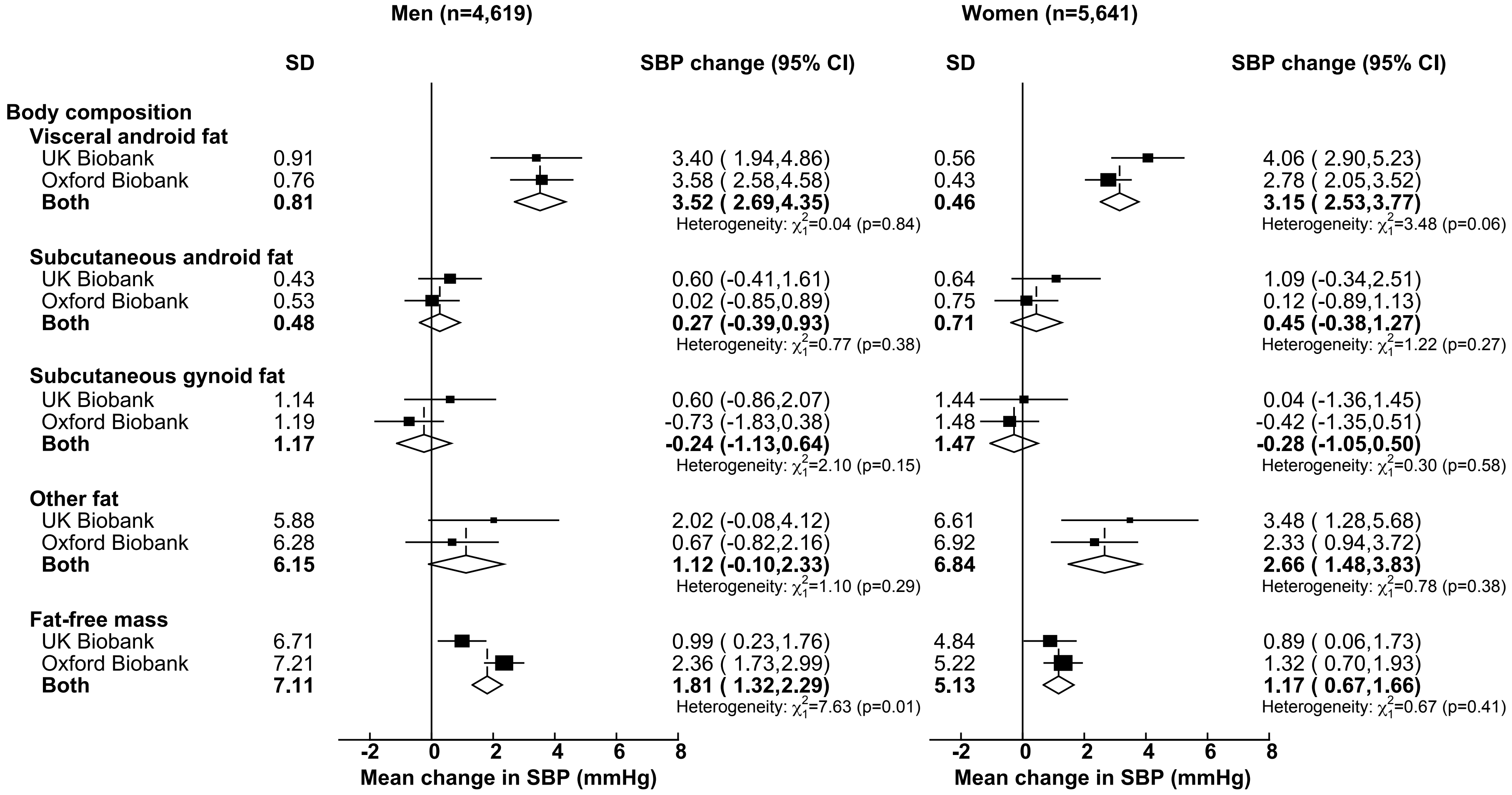

DXA = Dual energy X-ray Absorptiometry. Results presented as the mean change in SBP per standard deviation (SD) increase in DXA-derived body composition variable, adjusted for age (5-year groups), height (sex-specific fifths), education/occupation (6 groups) and alcohol consumption (5 groups). Weighted average of the study-specific SDs, with weights equal to 1 divided by the variance of the study-specific beta coefficient. Exclusions as in Supplementary Table 1.

Figure S2. Mean change in systolic blood pressure (SBP) per 1 standard deviation (SD) higher DXA-derived body composition variables after further adjustment for smoking and physical activity, by study and sex

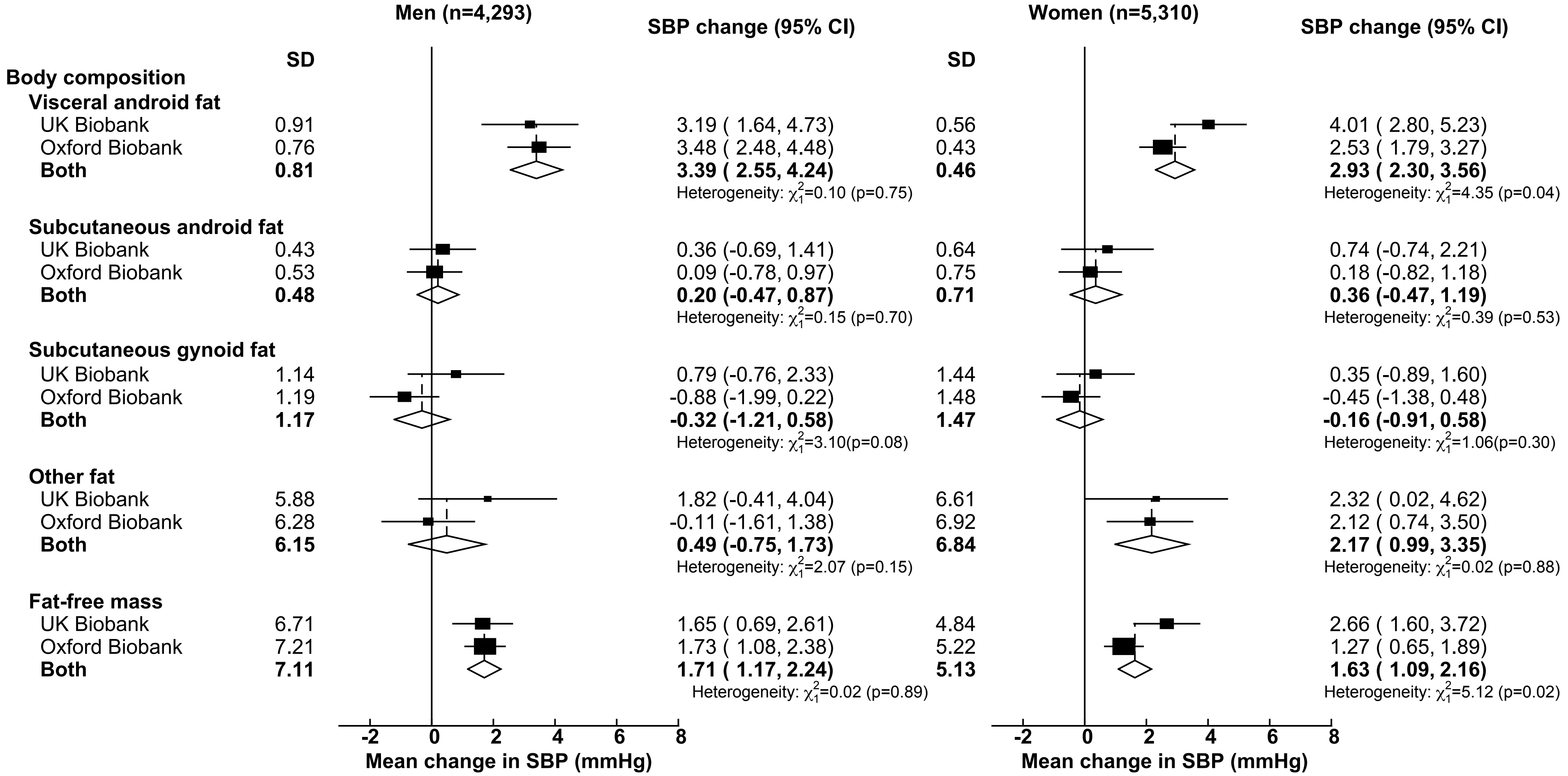

DXA= Dual energy X-ray Absorptiometry. Results presented as the mean change in SBP per standard deviation (SD) increase in DXA-derived body composition variable, adjusted for age (5-year groups), height (sex-specific fifths), education/occupation (6 groups), alcohol consumption (5 groups), physical activity (5 groups) and smoking (4 groups). Weighted average of the study-specific SDs, with weights equal to 1 divided by the variance of the study-specific beta coefficient. Exclusions as in Supplementary Table 1, with further exclusion of those on blood pressure lowering medication.

Figure S3. Mean change in systolic blood pressure (SBP) per 1 standard deviation (SD) higher log-transformed DXA-derived body composition variables, by study and sex

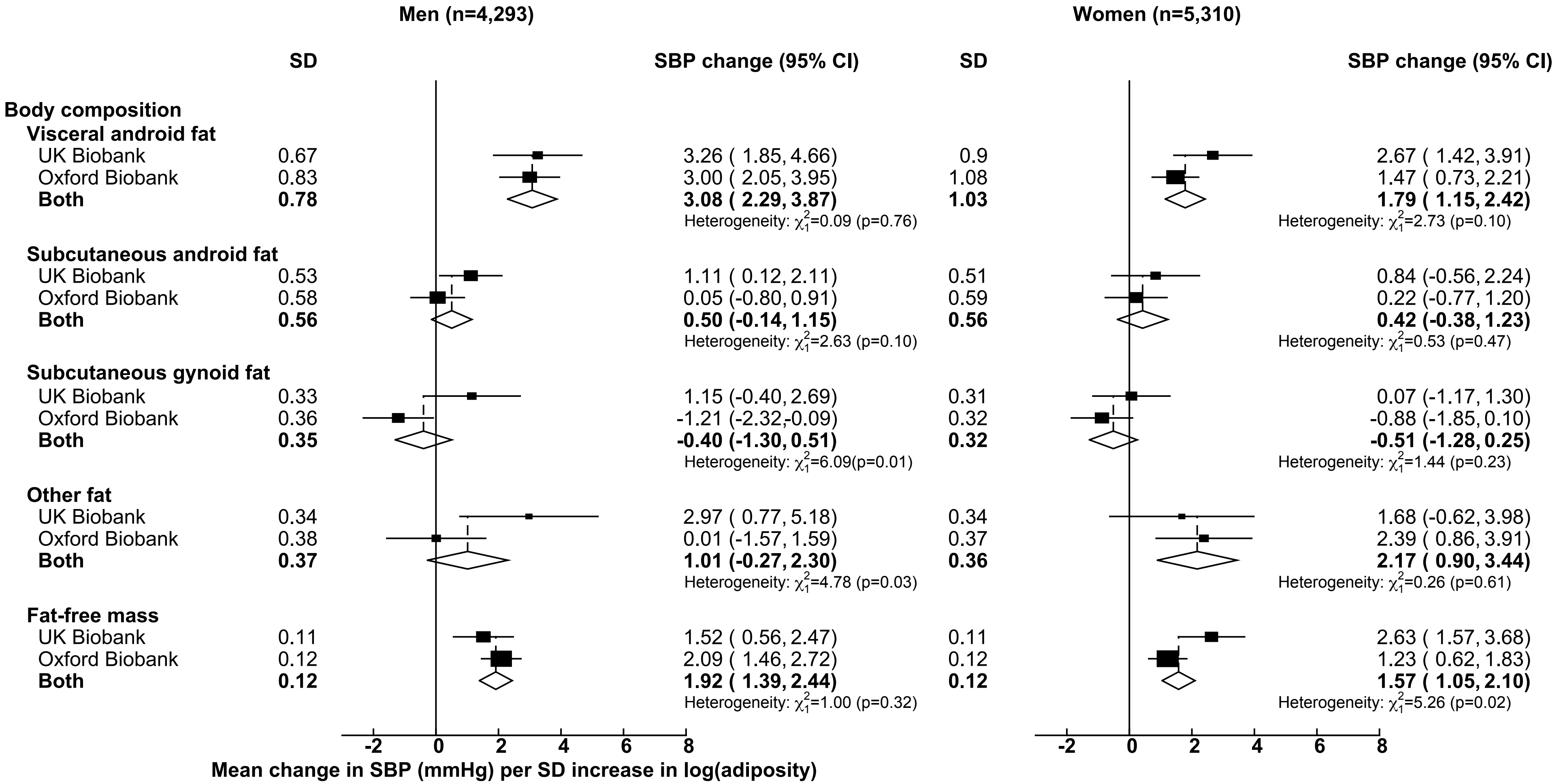

DXA= Dual energy X-ray Absorptiometry. Results presented as the mean change in SBP per standard deviation (SD) increase in the log-transformed DXA-derived body composition variable, adjusted for age (5-year groups), height (sex-specific fifths), education/occupation (6 groups) and alcohol consumption (5 groups). Weighted average of the study-specific SDs, with weights equal to 1 divided by the variance of the study-specific beta coefficient. Exclusions as in Supplementary Table 1, with further exclusion of those on blood pressure lowering medication.
